# Large thrombosed portomesenteric venous aneurysm treated with pharmacomechanical thrombolysis combined with TIPS placement

**DOI:** 10.1186/s42155-022-00288-0

**Published:** 2022-02-08

**Authors:** Ryan Kohlbrenner, Adam B Schwertner, Alexander R Vogel, Miles Conrad, R Peter Lokken

**Affiliations:** 1 University of California - San Francisco, 505 Parnassus Ave, M361, CA 94143 San Francisco, USA; 2 Denver Health and Hospital Authority, University of Colorado - Denver, 660 N. Bannock St., Pavilion L, Floor 1, 80204 Denver, CO United States; 3 Renown Regional Medical Center, Reno Radiological Associates, 1155 Mill St, 89502 Reno, NV United States

**Keywords:** Portal vein, Mesenteric veins, Splanchnic circulation, Aneurysm, TIPS, Thrombectomy, Thrombosis

## Abstract

**Background:**

Aneurysms are rare anomalies of the portomesenteric venous system. Thrombotic complications of these lesions can lead to mesenteric venous ischemia and bowel infarction, potentially requiring surgical intervention. Herein we describe a case of mesenteric ischemia due to a large thrombosed portomesenteric aneurysm treated with endovascular techniques.

**Case presentation:**

A 37-year-old previously healthy male who presented with abdominal pain to his local emergency department was found to have a thrombosed 12.0 × 5.1 cm portomesenteric venous aneurysm with evidence of mesenteric ischemia on CT. When conservative management with anticoagulation failed, transhepatic pharmacomechanical thrombolysis was initially performed. This was followed by TIPS placement with additional trans-TIPS thrombectomy to improve sluggish portal outflow and prevent re-thrombosis. The patient’s symptoms and imaging findings of ischemia resolved after endovascular therapy. No surgical intervention was required, and the patient was discharged on enoxaparin before being transitioned to apixaban. The TIPS remained patent at 2-year follow-up, with no change in the size of the aneurysm or re-thrombosis noted. The patient’s synthetic liver function was preserved with no evidence of hepatic encephalopathy during the follow-up period.

**Conclusions:**

Endovascular therapies may be used to manage thrombotic complications of portomesenteric venous aneurysms, obviating the need for surgical intervention in selected patients.

## Background

Portomesenteric aneurysms are uncommon abnormalities of the portal venous system that may be asymptomatic. Many can be attributed to portal hypertension and/or cirrhosis, pancreatitis, or prior surgery, but others have no identifiable cause and are present in otherwise healthy patients (Koc et al. 2007). Reported complications include portal vein thrombosis, portal vein rupture, and duodenal or biliary tract compression. While asymptomatic patients may be managed conservatively, surgical aneurysmorraphy or aneurysmectomy may be pursued for symptomatic aneurysms (Cho et al. 2008). A hybrid approach using both surgical and endovascular techniques has also been described in the setting of aneurysmal thrombosis (Gorolay et al. 2021). The case presented herein describes a patient with an acutely thrombosed complex portomesenteric aneurysm managed with endovascular techniques alone, sparing him a surgical intervention.

## Case presentation

**Fig. 1:**
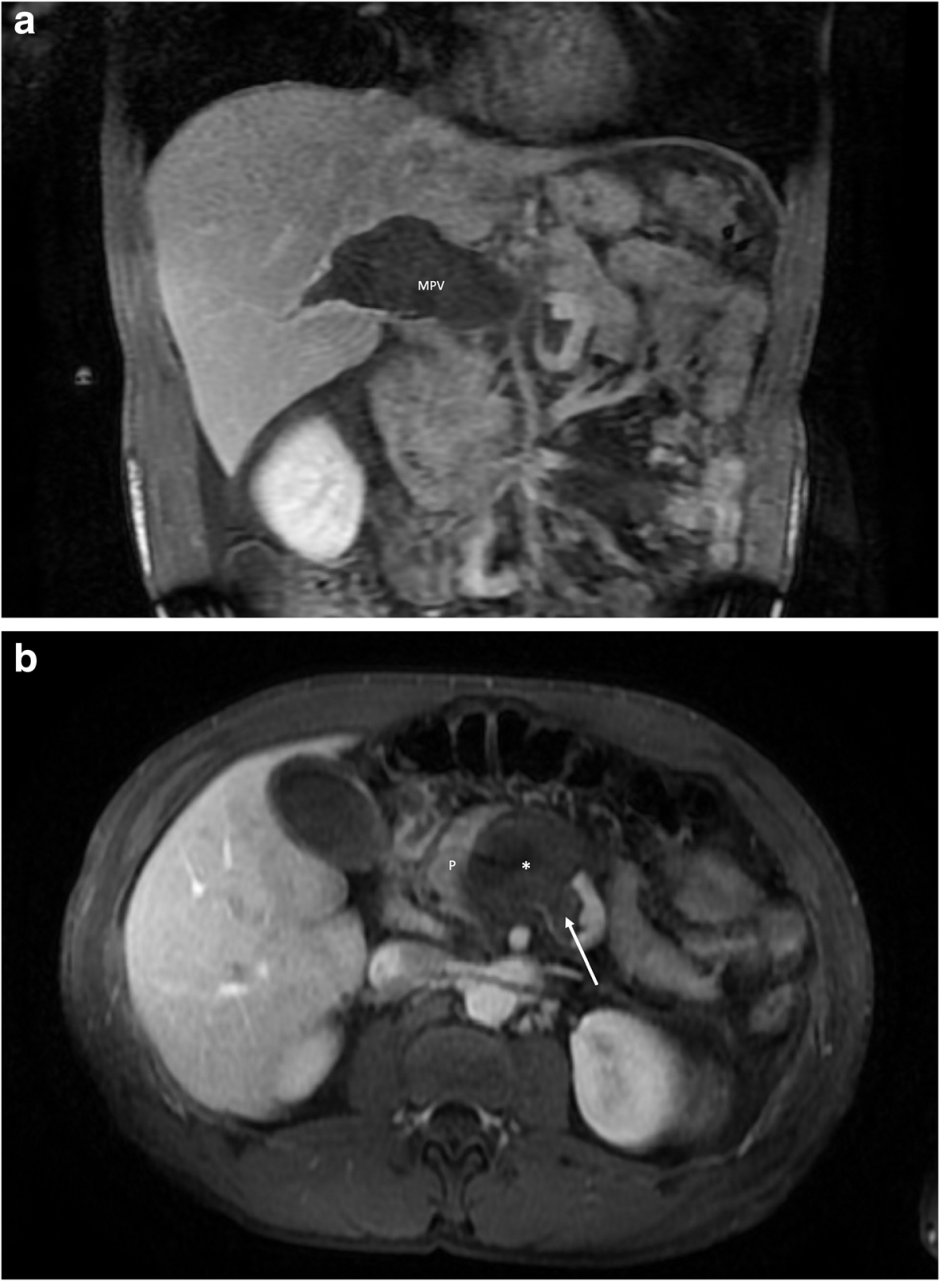
**A** Coronal contrast-enhanced T1 fat-saturated MRI image demonstrates the thrombosed extrahepatic portal venous component (MPV) of the large portomesenteric aneurysm. Thrombus also extends into the right portal vein. **B** Axial contrast-enhanced T1 fat-saturated MRI image demonstrates occlusive thrombus at the aneurysmal portosplenic confluence (*) abutting the adjacent pancreas (P). Non-occlusive thrombus is present in the central aspect of the splenic vein (arrow)

The patient is a non-cirrhotic 37-year-old previously healthy Caucasian male who presented to an outside facility with severe right upper quadrant and epigastric pain for three days. In the two weeks preceding the acute pain, he noted vague abdominal discomfort. The patient reported no significant medical or surgical history, fevers, medication use, recent dehydration, or alcohol abuse. On admission to the outside facility, bloodwork was notable only for a total bilirubin of 1.7 mg/dl. Serum albumin, AST, ALT, alkaline phosphatase, INR, ESR, and lipase were normal. White blood cell and platelet counts were within normal limits. Viral serologies were unrevealing and ethanol metabolite testing was negative. Tenderness to palpation was noted in the epigastrium and right upper quadrant. Portal venous phase CT of the abdomen and pelvis performed in the emergency department demonstrated a thrombosed fusiform aneurysm of the main portal vein (MPV) measuring 12.0 × 5.1 cm (Fig. 1A-B). The aneurysm was noted to involve the central aspects of the superior mesenteric vein (SMV) and splenic vein (SV). All intrahepatic portal veins, which were non-aneurysmal, were also thrombosed. The segmental and non-aneurysmal SMV branches were patent, as was the non-aneurysmal peripheral segment of the splenic vein. Mesenteric edema was noted, though bowel enhancement was present. Subsequent abdominal MRI with a T1 fat-saturated dynamic contrast enhanced sequence excluded an underlying portal vein tumor thrombus and an arteriovenous shunt. The liver parenchyma appeared normal without signs of cirrhosis. There was no splenomegaly or varices to suggest chronic portal hypertension. The patient’s ducts were non-dilated, and the arteries appeared normal without signs of an underlying vasculitis.

The patient was initially managed conservatively on intravenous heparin titrated to therapeutic PTT, as his segmental SMV branches remained patent. However, no improvement in clot burden or mesenteric edema was noted on portal venous phase CT after three days of anticoagulation. Given the persistent mesenteric congestion in the setting of intractable abdominal pain, endovascular management was pursued. Transhepatic access through the thrombosed left portal vein was achieved with a 21 G Chiba needle. The left portal vein was preferentially targeted, as it was easily visualized with real-time sonography and accessible with a single puncture. After wire passage, the access was upsized with a conversion set (AccuStick; Boston Scientific; Marlborough, MA) and a 6 Fr vascular sheath (Terumo Medical; Somerset, NJ) was placed. The main portal vein clot was easily traversed with a hydrophilic wire, and contrast venography was performed through a 5 Fr Kumpe catheter (Cook Medical; Bloomington, IN) in the SMV and splenic veins to delineate the margins of the thrombus. No flow was noted in the main portal vein. Rheolytic thrombectomy was then performed with 10 mg tissue plasminogen activator (tPA) through a 6 Fr pulse-spray catheter (AngioJet Solent; Boston Scientific). Clot maceration with 6 and 8mm angioplasty balloons in the central SMV and SV was also performed. As no significant improvement in flow was noted venographically, an ultrasound-accelerated thrombolysis catheter with 18 cm infusion length (EKOS; BTG; London, UK) was placed, spanning the clotted MPV and central SMV. tPA was administered overnight at 0.5 mg per hour, along with 800 units per hour of systemic heparin administered intravenously. The patient returned to the IR suite after approximately 24 h, where the infusion catheter was removed over a wire. Partial resolution of thrombus but persistent stasis of flow was noted on catheter portography. After upsizing the transhepatic sheath, additional rheolytic thrombectomy was performed with an 8 Fr pulse-spray catheter without adjuvant tPA (AngioJet ZelanteDVT; Boston Scientific). Due to persistent stagnation of contrast and extensive residual thrombus, a similar ultrasound-accelerated thrombolysis catheter with 18 cm infusion length was placed for overnight tPA infusion at 0.35 mg per hour with 800 units of systemic heparin administered intravenously.

**Fig. 2:**
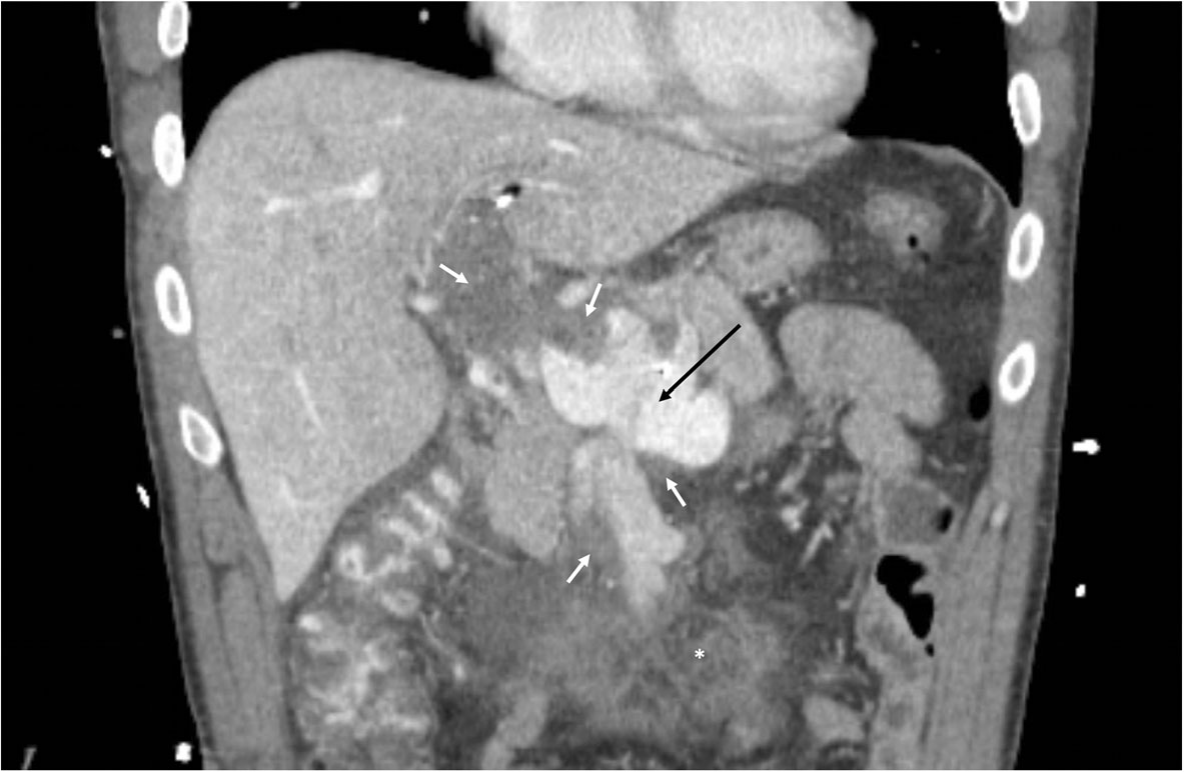
Coronal contrast-enhanced CT after thrombolysis and rheolytic thrombectomy. Although improvement is seen at the portosplenic confluence (black arrow), a large clot burden remains (white arrows). A transhepatic vascular sheath and thrombolysis catheter are partially visualized. Mesenteric edema (*) is also noted

**Fig. 3:**
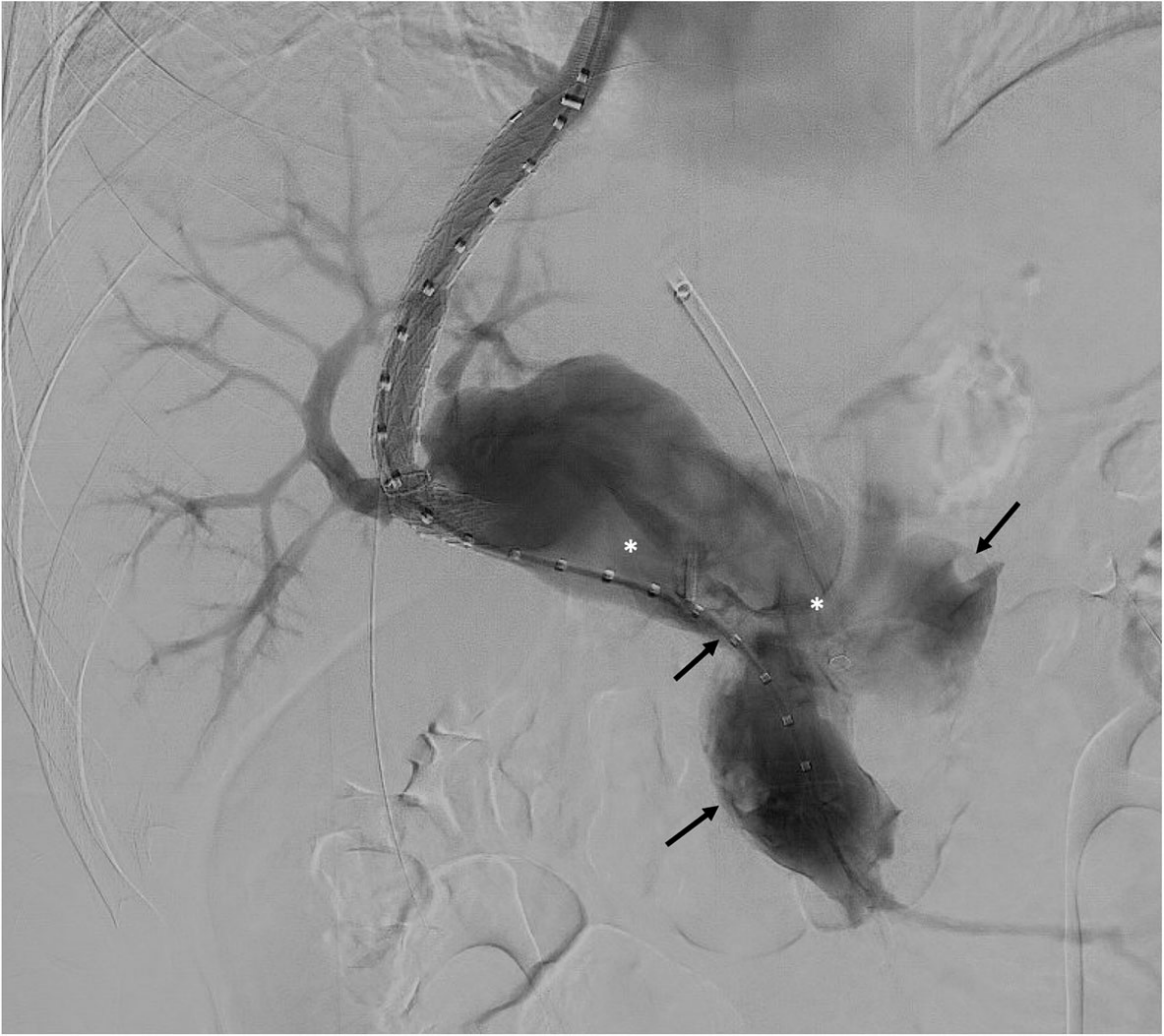
Portomesenteric venography after TIPS placement and additional rheolytic thrombectomy. Minimal non-occlusive thrombus remains (arrows). Turbulent flow of contrast through the aneurysm is noted (*). The posterior right and segment IV portal vein branches are now patent. The transhepatic vascular sheath within the occluded left portal vein is also visible

The patient was then transferred to a tertiary care facility for further management. While his abdominal pain had improved, he remained mildly tender to palpation on exam. An interval CT scan showed partial resolution of the thrombus within the aneurysm (Fig. 2) and recanalization of the segment IV and posterior right portal vein branches. As the risk of complete re-thrombosis was felt to be high, the multidisciplinary consensus was that a transjugular intrahepatic portosystemic shunt (TIPS) procedure would be necessary to create portal outflow. The procedure was performed under general anesthesia. After achieving right internal jugular venous access, an intracardiac echocardiogram (ICE) probe was used to guide a right hepatic to right posterior portal vein puncture, avoiding puncture of the aneurysm itself. Two needle passes were required before wire access into the thrombosed portal vein could be achieved. No resistance was noted during wire advancement, again suggesting the presence of acute thrombus. Portal pressures were not measured, given that elevated pressures would not distinguish between acute thrombosis and chronic portal hypertension, and that a TIPS was thought to be needed regardless. A 10mm TIPS endoprosthesis with an 8 cm covered segment (Viatorr CE; Gore Medical; Flagstaff, AZ) was placed and underdilated to 8mm, resulting in improved outflow on contrast portography. Hepatopedal flow was noted in the recanalized segment IV and posterior right portal vein branches (Fig. 3). To further reduce clot burden, additional rheolytic thrombectomy was performed under ICE and fluoroscopic guidance in regions of the large aneurysm not accessible by the transhepatic access. At the conclusion of the TIPS and thrombectomy, the existing 8 Fr left hepatic lobe sheath was removed with coil and gelatin sponge tract embolization. Full heparinization was resumed after the procedure. Given the improvement in flow through the aneurysm, additional thrombolysis was felt to be unnecessary.

**Fig. 4:**
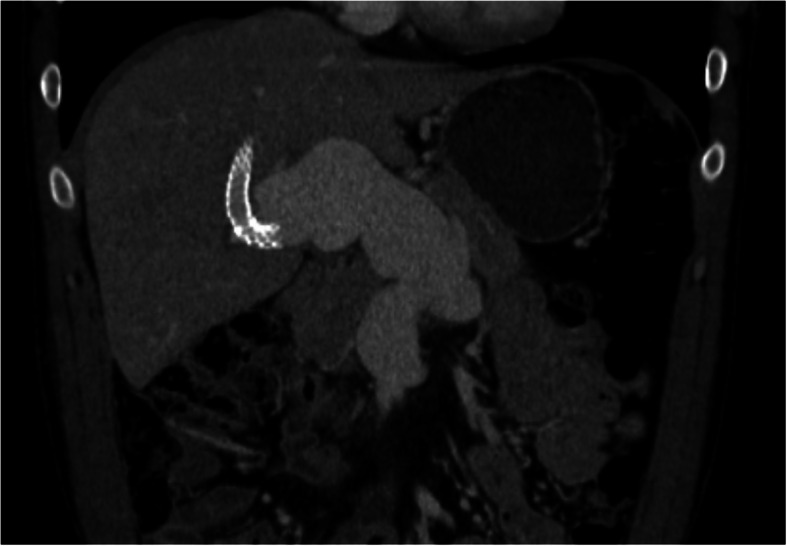
Coronal contrast-enhanced CT showing the widely patent portomesenteric aneurysm 1-month post-intervention. The right hepatic vein-to-right posterior portal vein TIPS is also patent

The patient was discharged pain-free six days later on enoxaparin as a bridge to oral warfarin therapy. He was transitioned to apixaban 5 mg twice-daily approximately 1 month after discharge. Additional workup for an inherited clotting disorder or vasculitis was negative. Biopsy was not pursued given absence of stigmata for chronic portal hypertension and normalization of his synthetic liver function after the episode. Follow-up CT scan 1 month later (Fig. 4) showed a patent TIPS and near-complete resolution of MPV, SMV, and SV thrombus. Complete resolution of the thrombus was evident on the 9-month follow-up MRI, with no interval change in the size of the aneurysm. Chronic left and anterior right portal vein thrombosis with peribiliary collaterals were noted. The TIPS and posterior right portal vein remained patent. The patient’s apixaban was reduced to 2.5 mg twice-daily at this time. Similar imaging findings were seen on a 2-year follow-up MRI, with a normal-appearing liver, patent TIPS, and no significant change in the size of the aneurysm. High T2 signal was noted in the periphery of the patent venous aneurysm, suggesting turbulent flow, coupled with a central flow void directed toward the TIPS and patent right portal vein.

No episodes of encephalopathy have occurred post-TIPS. As the patient maintains an active lifestyle, he was advised to temporarily stop the apixaban during periods of outdoor activity, allowing him to temporarily rely on the TIPS outflow alone to prevent aneurysm re-thrombosis. Given the absence of complications related to the TIPS or dose-reduced anticoagulant use during the follow-up period, indefinite TIPS maintenance and continuation of anticoagulation are planned. Annual ultrasounds of TIPS velocity will be pursued, coupled with yearly surveillance MRI to assess aneurysm size.

## Conclusions

This report describes endovascular treatment of a spontaneously thrombosed portomesenteric aneurysm, likely predisposed by its slow and turbulent flow. While surgical correction of these vascular lesions has been reported, there is no comparative data to suggest that outcomes are improved (Laurenzi et al. 2015). In this case, involvement of the superior mesenteric and splenic veins would have made surgical intervention more challenging. TIPS decompression of an extrahepatic portal venous aneurysm has been reported for a non-thrombotic lesion in the setting of portal hypertension (Dunlap et al. 2021). Given the success of TIPS placement and pharmacomechanical thrombectomy in our patient, minimally invasive therapies may also be used to manage thrombotic complications of these rare anomalies.

## Data Availability

Data sharing is not applicable to this article as no datasets were generated or analyzed during the current study.

## Abbreviations

ALT: alanine aminotransferase
AST: aspartate aminotransferase
CT: computed tomography
ESR: erythrocyte sedimentation rate
ICE: intracardiac echocardiogram
INR: international normalized ratio
MPV: main portal vein
MRI: magnetic resonance imaging
SMV: superior mesenteric vein
SV: splenic vein
TIPS: transjugular intrahepatic portosystemic shunt
tPA: tissue plasminogen activator
